# What Motivates Chinese Mothers’ Involvement in Adolescents’ Learning? Longitudinal Investigation on the Role of Mothers’ Expectations of Adolescents’ Family Obligations and Adolescents’ Academic Performance

**DOI:** 10.3390/bs13080632

**Published:** 2023-07-29

**Authors:** Zeyi Shi, Beiming Yang, Bin-Bin Chen, Xiaochen Chen, Yang Qu

**Affiliations:** 1 School of Education and Social Policy, Northwestern University, Evanston, IL 60208, USA; zeyi.shi@northwestern.edu (Z.S.); beiming@u.northwestern.edu (B.Y.); 2 Department of Psychology, Fudan University, Shanghai 200433, China; 3 Department of Psychology, Renmin University of China, Beijing 100086, China; xiaochenchen@ruc.edu.cn

**Keywords:** parental involvement in adolescents’ learning, parental expectation, family obligation, academic performance, culture, China

## Abstract

(1) Background: Parental involvement in adolescents’ learning generally benefits adolescents’ development, thus highlighting the importance of investigating why parents involve. Specifically, Chinese parents are highly involved in adolescents’ learning, which may be explained by their cultural beliefs. This longitudinal study provided a novel cultural understanding of the antecedents of Chinese mothers’ involvement in adolescents’ learning by examining the predicting effect of their expectations of adolescents’ family obligations over time, with attention to how adolescents’ academic performance moderated such effect. (2) Methods: Chinese mothers (*N* = 450; *M*_age_ = 39.52 years, *SD* = 3.96) of middle-school adolescents reported on their expectations of adolescents’ family obligations at Wave 1 and their involvement in adolescents’ learning twice over six months. Adolescents’ academic performance (i.e., grade) was obtained from teachers. (3) Results: Chinese mothers who had greater expectations of adolescents’ family obligations were involved more in adolescents’ learning over time. Moreover, adolescents’ academic performance moderated this longitudinal association, such that mothers’ expectations only predicted their greater involvement among adolescents with high, but not low, academic performance. (4) Conclusions: These findings highlight the cultural understanding of parents’ beliefs that motivate their involvement in adolescents’ learning in a non-Western society, as well as the moderating role of adolescents’ characteristics.

## 1. Introduction

Adolescence is a period when children may experience declines in academic motivation [1], which may further diminish their academic achievement [1]. However, parental involvement in adolescents’ learning, such as the time, money, and energy committed to adolescents’ schooling [2,3], could promote adolescents’ academic and non-academic functioning [2,4,5]. Given the significant implications of parental involvement in adolescents’ learning, it is especially of importance to examine what factors may contribute to it [6]. Notably, culturally guided beliefs in certain societies could create unique assets to parents that promote their involvement in children’s learning. For example, Chinese parents were found to be highly involved in children’s learning, compared to their counterparts in Western societies such as the United States [7]. In China, Confucian philosophy places great emphasis on children fulfilling family obligations toward their parents, such as respecting and repaying parents for raising them both materially and psychologically [8,9,10,11]. It is possible that Chinese parents’ expectations of adolescents to respect them and repay them (i.e., expectations of adolescents’ family obligations) may be one of the culturally guided beliefs that motivate them to be involved in adolescents’ learning over time. Moreover, adolescents’ characteristics, such as their academic performance, could also play a role in parents’ involvement in adolescents’ learning [6,12,13]. However, no study to date has examined whether Chinese parents’ expectations of adolescents’ family obligations contributed to their involvement in adolescents’ learning and whether adolescents’ academic performance might moderate the association between Chinese parents’ expectations and their involvement. Therefore, to address these research gaps, the current two-wave longitudinal research investigated the role of Chinese mothers’ expectations of adolescents’ family obligations in their involvement in adolescents’ learning over time and examined the moderating role of adolescents’ academic performance in this longitudinal association. Examining these research questions will not only shed light on cultural understanding of the motivating factors of parental involvement in adolescents’ learning but also bring attention to the intricate interplay between parental beliefs and adolescent factors that contribute to parental involvement.

### 1.1. Parental Involvement in Children’s Learning in China

Parental involvement in children’s learning is defined as parents’ commitment of resources to different contexts of children’s learning [2]. It includes various forms of involvement, such as home-based involvement (e.g., homework assistance), school-based involvement (e.g., attending school meetings), and academic socialization (e.g., communicating the importance of education) [2,5]. In past research, the positive implications of many forms of parental involvement for children’s and adolescents’ academic achievement have been established across different countries [2,4,5]. Moreover, beyond the academic arena, parental involvement in children’s and adolescents’ learning is also beneficial for their socioemotional adjustment [5]. This is because parents could help children develop skills, promote motivation in learning, and support children emotionally when they are involved [14,15]. Given that children’s academic motivation may start to decline during adolescence [1] and parental involvement in learning still plays a significant role during this period [5], it is particularly helpful to understand what sustains parental involvement in adolescents’ learning.

In particular, culture could create unique assets to parents that fosters their involvement in adolescents’ learning [16,17]. For example, prior research has suggested that Chinese parents tend to be highly involved in children’s learning [7]. Moreover, results from cross-cultural comparisons found Chinese parents to have heightened involvement in adolescents’ learning compared to their counterparts in the United States [7]. Such heightened involvement among Chinese parents might partially explain why Chinese adolescents were consistently found to outperform counterparts from other countries academically [18,19] and to preserve their academic motivation during adolescence [20]. However, these studies have mainly investigated the role of culture in parental involvement in adolescents’ learning through cross-cultural comparisons. It is especially of importance to “unpack culture” and investigate what culturally guided factors may contribute to Chinese parents’ involvement in adolescents’ learning beyond cross-cultural comparisons [16,17]. Thus, one of the major research questions of the present research is to examine what culturally guided beliefs endorsed by parents may play a role in Chinese parents’ involvement in adolescents’ learning.

### 1.2. Chinese Parents’ Expectations of Adolescents’ Family Obligations and Involvement in Adolescents’ Learning

Confucian ideologies place great emphasis on children fulfilling family obligations (e.g., respecting and following the wishes of parents and providing support to the family) toward their parents [11,21,22]. For example, filial piety, a key virtue in Confucian philosophy, underscores the importance of children repaying parents for the efforts in raising them, such that children should be the top students and support their elderly parents both materially and psychologically when they grow up [8,10,11]. Although the meaning behind filial piety has undergone changes during recent decades because of rapid social changes [23,24], the endorsement of family obligations is still prevalent in contemporary Chinese societies [25,26,27,28,29]. Moreover, fulfilling family obligations is often considered Chinese adolescents’ first step toward adulthood and maturity [30,31]. Guided by Confucian ideologies, Chinese parents usually hold high expectations of their adolescents’ family obligations so that their adolescents could respect them and support them financially and emotionally [32].

Chinese parents who expect adolescents to endorse family obligations may be highly motivated to be involved in adolescent learning. Based on the expectancy-value model of parents’ socialization of motivation [33], parents’ expectations for children are manifested in their parenting practices. Through parental involvement in adolescents’ learning, adolescents could develop the virtue of diligence and have better academic achievement that will bring honor and financial returns to the family [16,20], therefore fulfilling parents’ expectations of their family obligations. Moreover, Chinese parents’ commitment of time, energy, and money to adolescents’ learning may be at the cost of their personal interests or needs (e.g., personal recreation time) to fulfill adolescents’ needs in learning, thus being perceived by adolescents as parents’ sacrifice made for them [29]. Given the reciprocal nature of parent–child relationships in Chinese culture [11], parents’ motivation of involvement may be guided by their expectation for adolescents to obey and pay back to them (i.e., expecting adolescents to fulfill family obligations) in return for their sacrifice. Interestingly, prior research mainly focused on Chinese parents’ own family obligations (e.g., their sense of responsibility to their children in learning) or parents’ expectations of children’s educational attainment as antecedents of parents’ involvement in children’s learning [17,34,35]. However, no research to date has investigated the role of parents’ expectations of adolescents’ family obligations in their involvement in adolescents’ learning, especially in longitudinal research. Drawing on extant literature, the current research thus aims to address this serious research gap by examining whether Chinese parents’ expectations of adolescents’ family obligations may contribute to their involvement in adolescents’ learning over time. Examining this research question will extend cultural understanding of Chinese parents’ motivation to involve in adolescents’ learning.

### 1.3. The Moderating Role of Adolescents’ Academic Performance

In addition to the role of parental expectations of adolescents’ family obligations, adolescents’ own characteristics, such as their academic performance, may also contribute to parents’ involvement in adolescents’ learning [3,6,14]. It is possible that adolescents’ academic performance may moderate the association between parents’ expectations of adolescents’ family obligations and parental involvement in adolescents’ learning. Based on the expectancy-value model of parents’ socialization of motivation [33], children’s past academic performance may affect parents’ beliefs about how successful they can achieve certain outcomes (i.e., expectancy beliefs), that further influence their parenting practices. It is possible that parents who perceive adolescents’ poor academic performance may believe that it is unlikely for their adolescents to fulfill family obligations through learning even if they continue to be involved in much of children’s learning. Instead, they may commit their time, energy, and money to other aspects of adolescents’ development to help them fulfill family obligations (e.g., planning ahead for vocational schools, sports, or arts). Thus, the positive relations between Chinese parents’ expectations of adolescents’ family obligations and their involvement in adolescents’ learning might be weaker when their adolescents have poorer academic performance. One recent longitudinal study on Chinese parents indeed suggested that the poorer adolescents’ academic achievement was, the less parents were involved in adolescents’ learning a year later [13]. Unfortunately, past research only focused on the direct association between adolescents’ academic performance and parental involvement, instead of the interaction between the effect of parent expectations and adolescents’ academic performance [12,13,36]. No research has investigated whether adolescents’ academic performance may moderate the positive association between Chinese parents’ expectations of adolescents’ family obligations and their involvement in adolescents’ learning over time. Therefore, this study aims to tackle this serious research lacuna by investigating whether the longitudinal relations between Chinese parents’ expectations of adolescents’ family obligations and their involvement in adolescents’ learning may depend on adolescents’ academic performance. Such an investigation will provide insights into understanding how the effects of parents’ beliefs and adolescents’ own characteristics may interplay in explaining parents’ motivation to be involved in adolescents’ learning. 

### 1.4. Overview of the Current Research

**Figure 1:**
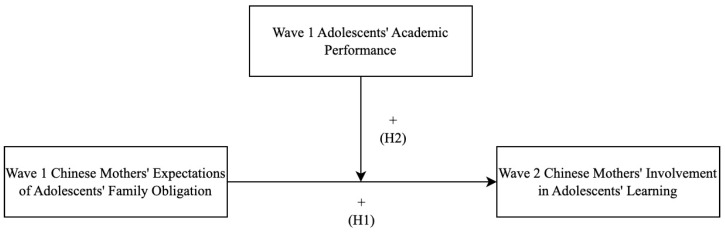
Proposed conceptual model in the current research. H1 = Hypothesis 1. H2 = Hypothesis 2. + indicates positive path coefficients. The model adjusted for mothers’ involvement in adolescents’ learning at Wave 1 and demographic covariates, which were not shown for clarity of presentation.

Parental involvement in children’s learning is crucial in helping children succeed during adolescence [2], which warrants investigation into the factors that contribute to parental involvement. Although prior research has suggested the importance of culturally guided parental beliefs and adolescents’ characteristics in motivating parents’ involvement in adolescents’ learning [16,17], no research has as directly examined whether Chinese parents’ expectations of adolescents’ family obligations guided by Confucian ideologies may play a role in their involvement in adolescents’ learning over time. In addition, it is unknown whether adolescents’ academic performance may influence such longitudinal association between parental expectations and their involvement. Therefore, the current two-wave longitudinal research aimed to investigate the longitudinal relations from Chinese mothers’ expectations of adolescents’ family obligations to their involvement in adolescents’ learning, with attention to the moderating role of adolescents’ academic performance in such longitudinal relation (for a conceptual model, see Figure 1).

To this end, Chinese mothers of seventh and eighth graders reported on their expectations of adolescents’ family obligations at Wave 1 as well as their involvement in adolescents’ learning at both Wave 1 and six months later at Wave 2. Adolescents’ academic performance at Wave 1 was obtained from schoolteachers. Two major hypotheses were proposed (see Figure 1). First, drawing on the expectancy-value model of parents’ socialization of motivation [33], as well as empirical evidence in Chinese culture [35], Chinese mothers’ expectations of adolescents’ family obligations were hypothesized to predict their increased involvement in adolescents’ learning over time (H1). Second, following the theoretical model of parental involvement [6] and expectancy-value theory [33], adolescents’ own academic performance was hypothesized to moderate the longitudinal relations between Chinese mothers’ expectations of adolescents’ family obligations and their involvement in adolescents’ learning (H2). Based on empirical evidence suggesting that Chinese parents were involved more when adolescents had better academic performance [13], it was hypothesized that Chinese mothers’ expectations of adolescents’ family obligations might be more likely to predict their involvement in adolescents’ learning longitudinally when adolescents had high (vs. low) academic performance.

## 2. Materials and Methods

### 2.1. Participants

Participants were 450 mothers (*M*_age_ = 39.52 years and *SD* = 3.96) of adolescents (*M*_age_ = 13.78 years and *SD* = 0.71, 49.1% girls) in seventh and eighth grade at the beginning of the project. Participants resided in Huzhou, which is a prefecture-level city in the northern Zhejiang province of Eastern China with a population of approximately 2.64 million [37]. Compared to large cities such as Shanghai and Beijing, Huzhou has significantly lower GDP per capita (about 30% lower), which is closer to the national average [37]. Adolescents attended an average-achieving middle school, although there was variability in adolescents’ achievement within the school. In regard to mothers’ highest level of educational attainment, 61.7% of mothers had at least a middle school diploma; 23.2% of mothers had a high school diploma; and 15.1% of mothers had education beyond high school (e.g., a bachelor’s or master’s degree).

### 2.2. Procedure

Ethical approval for the study was obtained from the Ethics Committee of the Department of Psychology, Renmin University of China, and mothers and their adolescents provided their written consent prior to data collection. Data were obtained from a longitudinal study focused on middle school Chinese adolescents’ family relationships and adjustment. Data from each wave were approximately six months apart. At Wave 1 and six months later at Wave 2, mothers completed questionnaires at home. Adolescents’ academic performance at Wave 1 was obtained from teachers. The attrition rate from Wave 1 to Wave 2 was 22.89%. The comparison of mothers completing both waves to those completing only the first revealed no differences at Wave 1 on any of the variables examined in this report (*F*s < 2.96 and *p*s > 0.09). Adolescents received small gifts (i.e., a notebook and a pencil) for their family’s participation. 

### 2.3. Measures

#### 2.3.1. Mothers’ Involvement in Adolescents’ Learning

Mothers’ involvement in adolescents’ learning at Wave 1 and Wave 2 was assessed with thirteen items (see Appendix A) adapted from prior research [38,39]. At both waves, mothers reported on how often (1 = never and 3 = always) they involved themselves in adolescents’ learning, in terms of school-based involvement (e.g., “I attend parent-teacher conferences”), home-based involvement (e.g., “I help with homework”), and academic socialization (e.g., “I talk to my child about what he/she is learning in school”). Two sets of Confirmatory Factor Analyses (CFA) were performed to test the three-factor model (i.e., school-based involvement, home-based involvement, and academic socialization) of mothers’ involvement in adolescents’ learning at both waves. The models had acceptable goodness of fit at both waves [40,41] (χ^2^ s < 175.088, CFIs > 0.926, TLIs > 0.902, and RMSEAs < 0.069), supporting the three-factor structure of mothers’ involvement in adolescents’ learning. The means of the items from each type of involvement were taken at Wave 1 (αs = 0.62 to 0.81) and Wave 2 (αs = 0.64 to 0.81) with higher numbers reflecting greater involvement of this certain type. The three types of involvement were positively correlated at Wave 1 (*r*s = 0.38 to 0.48 and *p*s < 0.001) and Wave 2 (*r*s = 0.45 to 0.49 and *p*s < 0.001) and were thus used as indicators of the latent construct of mothers’ involvement in adolescents’ learning in the central analyses following prior research [35]. 

#### 2.3.2. Mothers’ Expectations of Adolescents’ Family Obligations

To assess mothers’ expectations of adolescents’ family obligations at Wave 1, the measure that was used to assess adolescents’ feelings of obligation toward parents was adapted [30,42]. Mothers indicated how much (1 = not at all and 5 = very much) they expected their adolescents to engage in the activity described from nine items (see Appendix B), in terms of adolescents’ respect for the family, current assistance, and future support (e.g., “Respect parents” and “Help parents financially when they get older”). The result from CFA of mothers’ expectations of adolescents’ family obligations indicated good overall fit for the one-factor model [40,41] (χ^2^(15) = 47.168; CFI = 0.985; TLI = 0.965; and RMSEA = 0.069). The mean of the items was taken, with a higher number representing mothers’ greater expectations of adolescents’ family obligations (α = 0.88).

#### 2.3.3. Adolescents’ Academic Performance

Adolescents’ academic performance at Wave 1 was assessed with their current grades in Chinese and English, which are two major subjects in school. Adolescents’ Chinese and English grades were obtained from schoolteachers. These grades were also standardized (*z* score) within the same grade level (Grade 7 and Grade 8). Given that adolescents’ Chinese and English grades were highly correlated with each other in both Grade 7 and Grade 8 (*r*s > 0.66 and *p*s < 0.001), the *z* scores of both subjects were averaged to yield a composite score for adolescents’ academic performance at Wave 1 for analyses. 

### 2.4. Data Analyses Plan

To investigate the main effect of mothers’ expectations of adolescents’ family obligations on their involvement in adolescents’ learning over time and examine whether adolescents’ academic performance moderated this association, two sets of path models were conducted under the framework of Structural Equation Modeling (SEM) using the lavaan package in R [43]. In the first set of SEM analyses, mothers’ expectations of adolescents’ family obligations at Wave 1 were modeled to predict the latent construct of their involvement in adolescents’ learning six months later at Wave 2, indicated by their school-based involvement, home-based involvement, and academic socialization.

**Figure 2:**
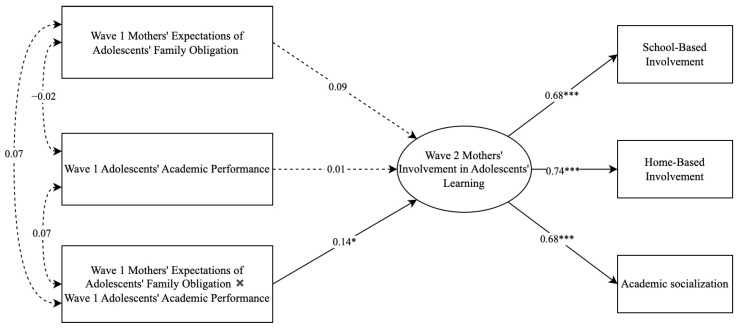
Structural equation modeling predicting mothers’ involvement in adolescents’ learning over time. Mothers’ expectations at Wave 1 were centered, and adolescents’ academic performance at Wave 1 was standardized within the same grade level before entering into the model. The model controlled for demographic covariates and mothers’ involvement in adolescents’ learning at Wave 1, which was also allowed to covary with the predictors but was not shown for clearer presentation. The standardized coefficients yielded by these analyses are presented. * *p* < 0.05. *** *p* < 0.001.

In the second set of SEM analyses (see Figure 2), adolescents’ academic performance at Wave 1 and the interaction term between adolescents’ academic performance and mothers’ expectations of adolescents’ family obligations at Wave 1 were further added to predict mothers’ involvement at Wave 2. Mothers’ expectations at Wave 1 were mean centered before entering into the model and generating the interaction term. A significant path from the interaction term at Wave 1 to parental involvement at Wave 2 indicates that adolescents’ academic performance moderated the effect of mothers’ expectations for adolescents’ family obligations on their involvement. Follow-up simple slope tests were then conducted to examine the longitudinal association between mothers’ expectations and involvement at low (i.e., 1 *SD* below the mean) and high (i.e., 1 *SD* above the mean) levels of adolescents’ academic performance. Both models controlled for mothers’ involvement in adolescents’ learning at Wave 1 and multiple demographic covariates, including adolescents’ age, grade, gender, and mothers’ educational attainment. Moreover, in both models, the error terms of all predictors were allowed to covary with each other.

Lastly, two sets of sensitivity analyses were conducted to examine whether the main effect of mothers’ expectations and the moderating effect of adolescents’ academic performance at Wave 1 on mothers’ involvement at Wave 2 were robust across the three types of maternal involvement in adolescents’ learning. The models were similar to the central analyses, except that the analyses predicted the observed variables of mothers’ school-based involvement, home-based involvement, and academic socialization at Wave 2 simultaneously, instead of the latent construct of mothers’ involvement. In addition, each set of SEM analyses included an unconstrained model (all paths to be freely estimated) and a more parsimonious constrained model (i.e., forcing the path coefficients indicating the main effects or the moderating effects to be equal across the three types of involvement). Nonsignificant χ^2^ change in the constrained model relative to the unconstrained model indicates that the models fit the data equally well and the strengths of the paths (i.e., main effects or moderating effects) were equal across the three types of involvement, and the more parsimonious constrained model would be adopted as the final model [41].

## 3. Results

### 3.1. Preliminary Analyses

Table 1 shows descriptive statistics and the zero-order correlations among the major variables. Mothers’ expectations of adolescents’ family obligations at Wave 1 were positively correlated with their involvement in adolescents’ learning at Wave 1 and six months later at Wave 2 (*r*s > 0.18 and *p*s < 0.001). Demographic covariates (i.e., adolescents’ age, grade, gender, and mothers’ educational attainment) were associated with adolescents’ academic performance at Wave 1 (|*r*|s > 0.19 and *p*s < 0.001). Mothers’ educational attainment was also positively correlated with their involvement in adolescents’ learning at Wave 1 and Wave 2 (*r*s = 0.15 and *p*s < 0.01).

### 3.2. Central Analyses

The first aim of the current research was to investigate whether Chinese mothers’ expectations of adolescents’ family obligations might play a role in their involvement in adolescents’ learning over time. The results indicated that in the first set of SEM analyses examining the longitudinal relations from mothers’ expectations of adolescents’ family obligations to their involvement in adolescents’ learning, the model had perfect fit because it was saturated, with the three types of involvement loaded well onto the latent construct of mothers’ involvement in adolescents’ learning (factor loadings > 0.68 and *p*s < 0.001). As expected, mothers who had stronger expectations for adolescents to have family obligations at Wave 1 would have increased involvement in adolescents’ learning six months later at Wave 2 (β = 0.12 and *p* = 0.048), over and above mothers’ initial involvement at Wave 1 and all demographic covariates (i.e., adolescents’ age, grade, gender, and mothers’ educational attainment). 

The second aim of the current research was to investigate whether the association between mothers’ expectations of adolescents’ family obligations and their involvement in adolescents’ learning over time might be dependent on the levels of adolescents’ academic performance. To this end, the second set of SEM analyses (see Figure 2) examined whether adolescents’ academic performance at Wave 1 moderated the longitudinal relations between mothers’ expectations of adolescents’ family obligations and their involvement in adolescents’ learning. The model also had perfect fit because it was saturated (factor loadings > 0.68 and *p*s < 0.001). The interaction term between mothers’ expectations of adolescents’ family obligations and adolescents’ academic performance at Wave 1 significantly predicted mothers’ involvement in adolescents’ learning at Wave 2, over and above mothers’ initial involvement and demographic covariates (β = 0.14 and *p* = 0.019), suggesting that adolescents’ academic performance significantly moderated the longitudinal relations between mothers’ expectations and their involvement.

**Figure 3:**
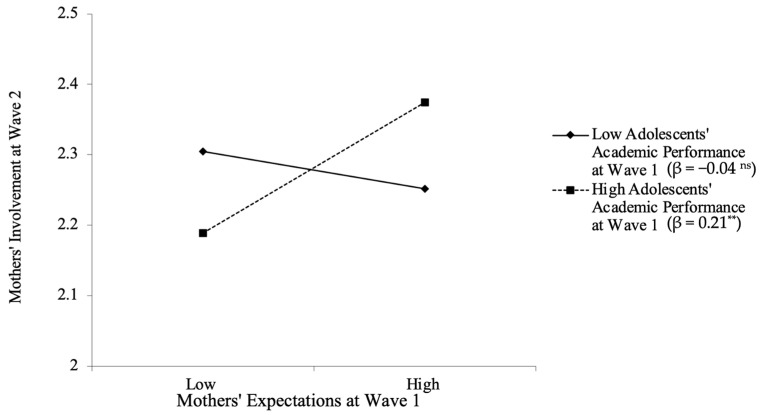
Adolescents’ academic performance moderated the longitudinal association between mothers’ expectations of adolescents’ family obligations and later involvement in adolescents’ learning. Mothers’ involvement at Wave 1 and demographic covariates (i.e., adolescents’ age, gender, grade, and mothers’ educational attainment) were controlled for in the analyses. Low and high adolescent academic performance (or mothers’ expectations of adolescents’ family obligations) are 1 *SD* below and above the mean. ** *p* < 0.01. ns = not significant.

To further decompose the moderating effect, simple slope tests were conducted to examine the longitudinal relations between mothers’ expectations of adolescents’ family obligations and later involvement in adolescents’ learning at high (i.e., 1 *SD* above mean) and low (i.e., 1 *SD* below mean) levels of adolescents’ academic performance. The results (see Figure 3) suggested that mothers’ expectations of adolescents’ family obligations only positively predicted their involvement in adolescents’ learning when adolescents had high (i.e., 1 *SD* above mean) levels of academic performance (β = 0.21 and *p* = 0.005). However, such an association was not evident when adolescents’ academic performance was low (i.e., 1 *SD* below mean) (β = −0.04 and *p* = 0.452). In summary, these results indicate that although mothers’ expectations of adolescents’ family obligations played a positive role in their involvement in adolescents’ learning over time, this association was only evident when adolescents had high levels of academic performance (i.e., 1 *SD* above mean).

To examine whether the main effect and moderating effect found in the central analyses were robust across the three types of parental involvement in adolescents’ learning (i.e., school-based involvement, home-based involvement, and academic socialization), two sets of sensitivity analyses in the context of SEM were further conducted. Results suggested that the constrained model (i.e., constraining the main effect of parents’ expectations on the three types of involvement to be equal) and unconstrained model fit the data equally well in main effect models (χ^2^ s < 9.76; CFIs > 0.994; TLIs > 0.972; and RMSEAs < 0.035), with no significant change in model fit (∆χ^2^(2) = 1.30 and *p* = 0.521). This result indicates that the positive role of mothers’ expectations in their involvement in adolescents’ learning was similar across the three types of involvement over time. Specifically, the greater mothers’ expectations of adolescents’ family obligations at Wave 1, the more school-based involvement, home-based involvement, and academic socialization they would have over time at Wave 2 (βs = 0.09 and *p*s = 0.02). In addition, the constrained model (i.e., constraining the interaction effect between adolescents’ academic performance and parents’ expectations on the three types of involvement to be equal) and unconstrained model also fit the data equally well in moderating effect models (χ^2^ s < 12.35; CFIs > 0.988; TLIs > 0.952; and RMSEAs < 0.041), with no significant change in model fit (∆χ^2^(2) = 3.11 and *p* = 0.211). This result indicated similar interactions between mothers’ expectations and adolescents’ academic performance in predicting the three types of maternal involvement over time (βs = 0.09 to 0.10 and *p*s = 0.02). Further simple slope tests decomposing the moderating effects also suggested similar findings to the central analyses. Specifically, mothers’ expectations of adolescents’ family obligations at Wave 1 predicted their increased school-based involvement, home-based involvement, and academic socialization over time when adolescents had high academic performance (βs = 0.12 to 0.20 and *p*s < 0.04), but not when adolescents had low academic performance (βs = −0.05 to 0.02 and *p*s > 0.30). These results suggest that adolescents’ academic performance at Wave 1 played similar roles in the longitudinal associations between mothers’ and the three types of involvement in adolescents’ learning (i.e., school-based involvement, home-based involvement, and academic socialization). Specifically, mothers who held higher expectations of adolescents’ family obligations had increased school-based involvement, home-based involvement, and academic socialization over time, only when adolescents’ concurrent academic performance was high (i.e., 1 *SD* above mean). Taken together, these results suggest that the main effects and moderating effects were robust across the three types of maternal involvement in adolescents’ learning, namely school-based involvement, home-based involvement, and academic socialization.

## 4. Discussion

Parental involvement in adolescents’ learning could promote adolescents’ academic and non-academic functioning across many countries [2,4,5], which highlights the significance of examining the contributing factors of parental involvement [6]. Notably, Chinese mothers are usually found to be highly involved in adolescents’ academic life [7], which may be guided by their cultural beliefs about adolescents’ family obligations. However, there lacks enough empirical investigation on how these culturally guided beliefs motivate parents to be involved in adolescents’ learning over time [16]. Therefore, to extend cultural understanding of the contributing factors of parents’ involvement in adolescents’ learning, the current research focused on the longitudinal association between Chinese mothers’ expectations of their adolescents’ family obligations and their involvement in adolescents’ learning, with attention to the moderating role of adolescents’ academic performance in this association. The results indicated that Chinese mothers’ higher expectations of adolescents’ family obligations predicted their increased involvement in adolescents’ learning over time. In addition, the interaction effect between maternal expectations and adolescents’ academic performance was found, such that mothers’ expectations of their adolescents’ family obligations positively predicted their involvement only when adolescents had high, but not low, academic performance.

As expected, the positive longitudinal relation was found between Chinese mothers’ expectations of adolescents’ family obligations and mothers’ involvement in adolescents’ learning (indicated by school-based involvement, home-based involvement, and academic socialization), over and above mothers’ baseline involvement and demographic covariates. Such a finding is in line with the expectancy-value model of parents’ socialization of motivation [33], indicating that parents’ expectations of children may be manifested in their practices in children’s learning (e.g., commitment of time, energy, and money to children’s learning). More importantly, the current result extends knowledge of culturally guided parental beliefs that motivate parental involvement in adolescents’ learning [16,17], beyond prior findings on Chinese parents’ own family obligations and expectations of adolescents’ high educational attainment [34,35]. It is possible that parents who expect adolescents to fulfill family obligations (i.e., respect, obey, and help with parents) may try to involve in adolescents’ learning, so that adolescents will develop the virtue of diligence, have enhanced academic and non-academic adjustment [20], and repay the sacrifice parents have undergone for them in return [29,44], thus fulfilling parents’ expectations consequently. 

The current research also suggests that adolescents’ academic performance interacted with the effect of Chinese mothers’ expectations in contributing to parental involvement in adolescents’ learning over time. This finding supports theoretical models indicating that child characteristics could also contribute to parents’ involvement beyond parental effects [6,14] and extends prior findings that mainly focused on the direct effect of children’s academic performance on parental involvement [12,13,36]. Of note, this study suggests that Chinese mothers’ expectations of adolescents’ family obligations predicted their heightened involvement only among adolescents who had high, but not low, academic performance. One of the potential explanations of the current finding is that adolescents’ poor (vs. high) academic performance perceived by mothers might lead to mothers’ beliefs that their adolescents might be less likely to fulfill family obligations through learning (i.e., lower expectancy beliefs) even if they continue to commit resources to adolescents’ academic life [33], thus leading to their diminished involvement in adolescents’ learning. Moreover, when adolescents have poor academic performance, parents who are involved in their learning may become frustrated with and worried about their adolescents, which may lead to unconstructive practices accompanied by their involvement (e.g., controlling practices and hostility toward adolescents) that potentially hurt their parent–adolescent relationship and adolescents’ academic functioning [14,15]. Thus, it is likely that to avoid such negative consequences (e.g., parent–adolescent conflicts and adolescents’ poor academic achievement) that diminish the possibilities of adolescents fulfilling family obligations (e.g., obeying, respecting, and repaying parents), parents whose adolescents have poor academic performance may choose to be less involved in their learning. 

### 4.1. Theoretical and Practical Implications

It is of great value to investigate why parents are involved in adolescents’ learning, given its importance for adolescents’ academic and non-academic functioning across different countries [2,4,5]. Notably, prior studies have usually found that Chinese parents tend to be highly involved in adolescents’ learning compared to their counterparts in Western societies, which may be explained by their beliefs shaped by culture. However, empirical evidence on the association between Chinese parents’ culturally guided beliefs and their involvement in adolescents’ learning is less known [16]. Inspired by the notion that Chinese culture places great emphasis on adolescents respecting, obeying, and repaying parents (i.e., fulfilling family obligations) [9,21,22], the current research provided novel cultural understanding into the antecedents of Chinese parents’ involvement in adolescents’ learning. Specifically, this longitudinal research found for the first time that Chinese mothers’ expectations of adolescents’ family obligations predicted their heightened involvement in adolescents’ learning over time, above and beyond mothers’ baseline involvement and demographic variables. Moreover, the current research also generated novel findings indicating the moderating effect of adolescents’ academic performance in the longitudinal association between Chinese mothers’ expectations and their involvement, such that mothers’ expectations only predicted their involvement when adolescents had high but not low academic performance. This result not only highlights the role of adolescents’ characteristics in parental involvement in adolescents’ learning [6] but also underscores the interaction between the effect of parents’ beliefs and adolescents’ characteristics on parenting in adolescents’ academic arena. Practically, these findings could provide fruitful insights for interventions that aim to promote caregivers’ involvement in adolescents’ learning that ultimately promote adolescents’ development [45]. Future intervention programs could build family–school partnerships and pay special attention to both cultural (e.g., culturally guided beliefs and values) and child factors (e.g., children’s academic difficulties) to foster parental involvement in children’s learning. 

### 4.2. Limitations and Future Directions

Despite the methodological merits, such as applying longitudinal design, the current research has several limitations that point to directions for future research. First, this research only examined the relations between parents’ expectations and their involvement in adolescents’ learning among Chinese mothers. It remains unknown whether the relations between parental involvement and expectations may also exist among Chinese fathers. For instance, prior research suggests that fathers generally consider children’s family obligations as less important compared to mothers [46], which leads to the possibility that fathers’ expectations of adolescents’ family obligations may be less likely to be manifested in their involvement in adolescents’ learning. Therefore, it is important for future research to also investigate the relations between fathers’ involvement and their expectations of adolescents’ family obligations and compare the similarities and differences in mothers and fathers. 

Second, the current research followed mothers over children’s early adolescence with two time-points over six months, which leaves an open question about whether the current findings also apply over a longer time span (e.g., when children enter middle and late adolescence). As children move through middle and late adolescence, their schoolwork becomes more difficult for parents to understand [47]. When parents have low self-efficacy in helping children academically, they tend to become less involved in children’s learning [34]. However, parents’ expectations of adolescents’ family obligations may not necessarily decline as adolescents grow [48]. Thus, future studies can employ longitudinal designs with a longer time span to examine whether parents’ expectations of children’s family obligations still motivate their involvement in learning as children progress toward maturity.

Finally, due to Chinese parents’ high levels of involvement in children’s learning, the current research focused on the relationship between parents’ involvement and expected family obligations in mainland China. However, it will be interesting to examine whether such relations also emerge in other cultural groups that highly value family obligation. For example, recent research has suggested that immigrant Chinese parents (vs. local Chinese parents) may especially highlight the value of education because performing well in school could help children overcome barriers (e.g., discrimination) and promote upward mobility in the host society [49]. It is possible that immigrant Chinese parents’ expectation of adolescents’ family obligations may more strongly guide their involvement in adolescents’ learning so that they could highlight the importance of learning and enhance children’s academic performance and finally help adolescents fulfill family obligations. Therefore, exploring whether the effect of parents’ expectations of children’s family obligations may vary across different cultural groups that value family obligations will provide a more nuanced cultural understanding of parents’ motivations for their involvement in children’s learning.

## 5. Conclusions

Parental involvement in adolescents’ learning is generally found to be promotive of adolescents’ academic and non-academic functioning, which underscores the importance of investigating what factors may contribute to it. Culture could create unique assets in parents’ belief systems that foster their involvement in adolescents’ learning. Specifically, in Chinese culture, Confucian ideologies place strong emphasis on adolescents fulfilling family obligations. However, research is lacking regarding whether Chinese parents’ expectations of adolescents’ family obligations may play a positive role in their involvement in adolescents’ learning over time. Moreover, adolescents’ own characteristics could also contribute to how much parents are involved in adolescents’ learning. Yet, no research has investigated whether adolescents’ academic performance may moderate the longitudinal association between Chinese parents’ expectations about adolescents’ family obligations and their involvement in adolescents’ learning. Using a two-wave longitudinal design, the current research suggests that Chinese mothers’ greater expectations of adolescents to fulfill family obligations are predictive of their heightened involvement in adolescents’ learning over time. More importantly, mothers’ expectations only predict their increased involvement among adolescents with high, but not low, academic performance. Taken together, the current research provides a unique cultural understanding of parents’ beliefs that motivate their involvement in adolescents’ learning and underscores the role of adolescents’ characteristics in such process. Drawing on these findings, intervention programs could pay special attention to the cultural assets in parents’ belief systems and consider the interaction between parents and adolescents to foster parental involvement in adolescents’ learning.

## Figures and Tables

**Table 1:** Descriptive statistics and correlations of variables.

Variables	1	2	3	4	5	6	7	8
1. Adolescents’ Academic Performance at Wave 1	--							
2. Mothers’ Expectation of Adolescents’ Family Obligations at Wave 1	−0.03	--						
3. Mothers’ Involvement in Adolescents’ Learning at Wave 1	0.04	0.21 ***	--					
4. Mothers’ Involvement in Adolescents’ Learning at Wave 2	0.04	0.18 ***	0.45 ***	--				
5. Adolescents’ Age	−0.20 ***	0.02	0.03	0.02	--			
6. Adolescents’ Grade Level	−0.22 ***	0.04	0.01	0.02	0.68 ***	--		
7. Adolescents’ Gender	0.26 ***	−0.01	0.03	0.04	−0.04	0.02	--	
8. Mothers’ Educational Attainment	0.19 ***	−0.02	0.15 **	0.15 **	−0.06	−0.08	0.09	--
Mean	76.47	4.24	2.25	2.28	13.78	7.40	1.49	2.37
*SD*	15.52	0.59	0.39	0.41	0.71	0.49	0.50	0.89

Note: For adolescents’ gender, 1 = boys, and 2 = girls. For mothers’ education, 1 = elementary school or lower, and 4 = college degree or higher. ** *p* < 0.01. *** *p* < 0.001.

## Data Availability

The data presented in this study are available on request from the corresponding authors.

## Appendix A

Items Measuring Mothers’ Involvement in Adolescents’ Learning

Home-based involvement

1. I help with homework.
2. I know how my child is doing in school.
3. I check to make sure my child’s homework is completed.
4. I enforce family rules or expectations about completing homework.

School-based involvement

5. I attend parent–teacher organization meetings.
6. I attend parent–teacher conferences.
7. I volunteer at my child’s school.
8. I watch my child in sports or school activities.

Academic socialization

9. I talk to my child about what he/she is learning in school.
10. I help my child select which courses to take (e.g., tutoring class).
11. I talk with my child about how his/her courses in school will prepare him/her for future careers.
12. I talk with my child about his/her educational plans for the future.
13. I help my child select which courses to take (e.g., extracurricular interest class).

## Appendix B

Items Measuring Mothers’ Expectations of Adolescents’ Family Obligation

1. Spend time at home with parents.
2. Spend holidays with parents.
3. Help with housework that parents need done.
4. Respect parents.
5. Obey parents.
6. Please parents.
7. Look after parents when they get older.
8. Help parents financially when they get older.
9. Stay in contact with parents when they get older.

## References

[1] Wigfield A., Eccles J.S. (2020). 35 Years of Research on Students’ Subjective Task Values and Motivation: A Look Back and a Look Forward. Advances in Motivation Science.

[2] Hill N.E., Tyson D.F. (2009). Parental Involvement in Middle School: A Meta-Analytic Assessment of the Strategies That Promote Achievement. Dev. Psychol..

[3] Grolnick W.S., Pomerantz E.M. (2022). Should Parents Be Involved in Their Children’s Schooling?. Theory Pract..

[4] Kim S. (2020). won Meta-Analysis of Parental Involvement and Achievement in East Asian Countries. Educ. Urban Soc..

[5] Barger M.M., Kim E.M., Kuncel N.R., Pomerantz E.M. (2019). The Relation between Parents’ Involvement in Children’s Schooling and Children’s Adjustment: A Meta-Analysis. Psychol. Bull..

[6] Hoover-Dempsey K.V., Walker J.M.T., Sandler H.M., Whetsel D., Green C.L., Wilkins A.S., Closson K. (2005). Why Do Parents Become Involved? Research Findings and Implications. Elem. Sch. J..

[7] Pomerantz E.M., Ng F.F.-Y., Cheung C.S.-S., Qu Y. (2014). Raising Happy Children Who Succeed in School: Lessons from China and the United States. Child Dev. Perspect..

[8] Chao R.K., Tseng V. (2002). Parenting of Asians. Handbook of Parenting: Volume 4 Social Conditions and Applied Parenting.

[9] Qu Y., Pomerantz E.M., Wang M., Cheung C., Cimpian A. (2016). Conceptions of Adolescence: Implications for Differences in Engagement in School over Early Adolescence in the United States and China. J. Youth Adolesc..

[10] Wang Q., Bornstein M.H. (2018). Filial Piety. The SAGE Encyclopedia of Lifespan Human Development.

[11] Bedford O., Yeh K.-H. (2021). Evolution of the Conceptualization of Filial Piety in the Global Context: From Skin to Skeleton. Front. Psychol..

[12] Tunkkari M., Aunola K., Hirvonen R., Silinskas G., Kiuru N. (2021). The Quality of Maternal Homework Involvement: The Role of Adolescent and Maternal Factors. Merrill-Palmer Q..

[13] Xiong Y., Qin X., Wang Q., Ren P. (2021). Parental Involvement in Adolescents’ Learning and Academic Achievement: Cross-Lagged Effect and Mediation of Academic Engagement. J. Youth Adolesc..

[14] Pomerantz E.M., Kim E.M., Cheung C.S.-S., Harris K.R., Graham S., Urdan T., Graham D., Royer J.M., Zeidner M. (2012). Parents’ Involvement in Children’s Learning. APA Educational Psychology Handbook, Vol 2: Individual Differences and Cultural and Contextual Factors.

[15] Pomerantz E.M., Grolnick W.S., Elliot A.J., Dweck C.S., Yeager D.S. (2017). The Role of Parenting in Children’s Motivation and Competence: What Underlies Facilitative Parenting?. Handbook of Competence and Motivation: Theory and Application.

[16] Ng F.F.-Y., Wei J. (2020). Delving into the Minds of Chinese Parents: What Beliefs Motivate Their Learning-Related Practices?. Child Dev. Perspect..

[17] Yamamoto Y., Li J., Bempechat J. (2022). Reconceptualizing Parental Involvement: A Sociocultural Model Explaining Chinese Immigrant Parents’ School-Based and Home-Based Involvement. Educ. Psychol..

[18] Mullis I.V.S., Martin M.O., Foy P., Kelly D.L., Fishbein B. (2020). TIMSS 2019 International Results in Mathematics and Science.

[19] OECD (2019). PISA 2018 Results (Volume i): What Students Know and Can Do.

[20] Cheung C.S.-S., Pomerantz E.M. (2015). Value Development Underlies the Benefits of Parents’ Involvement in Children’s Learning: A Longitudinal Investigation in the United States and China. J. Educ. Psychol..

[21] Ikels C. (2004). Filial Piety: Practice and Discourse in Contemporary East Asia.

[22] Fuligni A.J., Zhang W. (2004). Attitudes toward Family Obligation among Adolescents in Contemporary Urban and Rural China. Child Dev..

[23] Way N., Okazaki S., Zhao J., Kim J.J., Chen X., Yoshikawa H., Jia Y., Deng H. (2013). Social and Emotional Parenting: Mothering in a Changing Chinese Society. Asian Am. J. Psychol..

[24] Yeh K.-H., Yi C.-C., Tsao W.-C., Wan P.-S. (2013). Filial Piety in Contemporary Chinese Societies: A Comparative Study of Taiwan, Hong Kong, and China. Int. Sociol..

[25] Lin J.-P., Yi C.-C. (2013). A Comparative Analysis of Intergenerational Relations in East Asia. Int. Sociol..

[26] Wang Y.C. (2014). In Search of the Confucian Family: Interviews with Parents and Their Middle School Children in Guangzhou, China. J. Adolesc. Res..

[27] Cheah C.S.L., Leung C.Y.Y., Bayram Özdemir S. (2018). Chinese Malaysian Adolescents’ Social-Cognitive Reasoning Regarding Filial Piety Dilemmas. Child Dev..

[28] Chen X. (2020). Exploring Cultural Meanings of Adaptive and Maladaptive Behaviors in Children and Adolescents: A Contextual-Developmental Perspective. Int. J. Behav. Dev..

[29] Leung J.T. (2020). Perceived Parental Sacrifice, Filial Piety and Hopelessness among Chinese Adolescents: A Cross-Lagged Panel Study. J. Adolesc..

[30] Pomerantz E.M., Qin L., Wang Q., Chen H. (2011). Changes in Early Adolescents’ Sense of Responsibility to Their Parents in the United States and China: Implications for Academic Functioning. Child Dev..

[31] Qu Y., Pomerantz E.M., Wang Q., Ng F.F.Y. (2020). Early Adolescents’ Stereotypes About Teens in Hong Kong and Chongqing: Reciprocal Pathways with Problem Behavior. Dev. Psychol..

[32] Qu Y., Pomerantz E.M., Deng C. (2016). Mothers’ Goals for Adolescents in the United States and China: Content and Transmission. J. Res. Adolesc..

[33] Eccles J.S., Wigfield A. (2020). From Expectancy-Value Theory to Situated Expectancy-Value Theory: A Developmental, Social Cognitive, and Sociocultural Perspective on Motivation. Contemp. Educ. Psychol..

[34] Wei J., Pomerantz E.M., Ng F.F.-Y., Yu Y., Wang M., Wang Q. (2019). Why Does Parents’ Involvement in Youth’s Learning Vary across Elementary, Middle, and High School?. Contemp. Educ. Psychol..

[35] Wu N., Hou Y., Wang Q., Yu C. (2018). Intergenerational Transmission of Educational Aspirations in Chinese Families: Identifying Mediators and Moderators. J. Youth Adolesc..

[36] Silinskas G., Kiuru N., Aunola K., Lerkkanen M.K., Nurmi J.E. (2015). The Developmental Dynamics of Children’s Academic Performance and Mothers’ Homework-Related Affect and Practices. Dev. Psychol..

[37] Huzhou Bureau of Statistics (2019). Huzhou Statistics Yearbook.

[38] Hill N.E., Witherspoon D.P., Bartz D. (2018). Parental Involvement in Education during Middle School: Perspectives of Ethnically Diverse Parents, Teachers, and Students. J. Educ. Res..

[39] Day E., Dotterer A.M. (2018). Parental Involvement and Adolescent Academic Outcomes: Exploring Differences in Beneficial Strategies across Racial/Ethnic Groups. J. Youth Adolesc..

[40] McDonald R.P., Ho M.H.R. (2002). Principles and Practice in Reporting Structural Equation Analyses. Psychol. Methods.

[41] Kline R.B. (2016). Principles and Practice of Structural Equation Modeling.

[42] Qu Y., Pomerantz E.M. (2015). Divergent School Trajectories in Early Adolescence in the United States and China: An Examination of Underlying Mechanisms. J. Youth Adolesc..

[43] Rosseel Y. (2012). Lavaan: An R Package for Structural Equation Modeling. R Package Version 0.5-15. J. Stat. Softw..

[44] Leung J.T.Y., Shek D.T.L. (2020). Parental Sacrifice, Filial Piety and Adolescent Life Satisfaction in Chinese Families Experiencing Economic Disadvantage. Appl. Res. Qual. Life.

[45] Smith T.E., Sheridan S.M., Kim E.M., Park S., Beretvas S.N. (2020). The Effects of Family-School Partnership Interventions on Academic and Social-Emotional Functioning: A Meta-Analysis Exploring What Works for Whom. Educ. Psychol. Rev..

[46] Su T.F., Costigan C.L. (2009). The Development of Children’s Ethnic Identity in Immigrant Chinese Families in Canada: The Role of Parenting Practices and Children’s Perceptions of Parental Family Obligation Expectations. J. Early Adolesc..

[47] Kim S., Fong V.L. (2013). How Parents Help Children with Homework in China: Narratives across the Life Span. Asia Pac. Educ. Rev..

[48] Dong X., Zhang M., Simon M.A. (2014). The Expectation and Perceived Receipt of Filial Piety among Chinese Older Adults in the Greater Chicago Area. J. Aging Health.

[49] Ng F.F., Sze I.N., Tamis-LeMonda C.S., Ruble D.N. (2017). Immigrant Chinese Mothers’ Socialization of Achievement in Children: A Strategic Adaptation to the Host Society. Child Dev..

